# History of the implementation of public health emergency management in Brazil

**DOI:** 10.1590/S2237-96222024v34e20240498.en

**Published:** 2025-05-02

**Authors:** Ana Sara Semeão de Souza, João Roberto Cavalcante, Raquel Proença, Igor de Assis Rodrigues, Carlos Henrique Michiles Frank, Daniel Roberto Coradi de Freitas, Edenilo Baltazar Barreira, Ethel Leonor Maciel, Márcio Henrique de Oliveira Garcia

**Affiliations:** 1 Ministério da Saúde, Secretaria de Vigilância em Saúde e Ambiente, Brasília, DF, Brazil

**Keywords:** Public Health Surveillance, Surge Capacity, Brazilian National Health System, Declaration of Emergency, Vigilancia en Salud Pública, Capacidad de Reacción, Sistema Único de Salud, Declaración de Emergencia

## Abstract

Public health emergencies can cause significant social, economic and health impacts. To mitigate these effects, approaches and programs at national and global levels have been developed, focusing on preparedness, surveillance and response, especially for emergencies of international concern (1). As a Member State of the World Health Organization and signatory of its agreements and conventions, Brazil is committed to ensuring the execution of health actions with a focus on public health emergencies. Despite successful experiences in emergency management, this is still an emerging field, with limited scientific production. This article describes the history of public health emergency management at the federal level and its strategies for preparing, surveillance and responding to these events in the Brazilian National Health System.

## Resumen

Las emergencias de salud pública pueden causar impactos significativos en los ámbitos social, económico y sanitario. Para mitigar estos efectos, se han desarrollado enfoques y programas a nivel nacional y global, centrados en la preparación, la vigilancia y la respuesta, especialmente para emergencias de importancia internacional (1). Como Estado Miembro de la Organización Mundial de la Salud y signatario de sus acuerdos y convenciones, Brasil se compromete a garantizar la ejecución de acciones de salud con foco en emergencias de salud pública. A pesar de las experiencias exitosas en el manejo de emergencias, este es todavía un campo emergente, con producción científica limitada. Este artículo describe la historia de la gestión de emergencias de salud pública a nivel federal y sus estrategias para la preparación, vigilancia y respuesta ante estos eventos en el Sistema Único de Salud.

## Introduction

The National Health Foundation (2), created in 1991, played an essential role by integrating the National Epidemiology Center to promote the use of epidemiology in the formulation of health policies in the Brazilian National Health System (*Sistema Único de Saúde* - SUS). In the 1990s, emergency response was decentralized and depended on external support, such as that from the United States Centers for Disease Control and Prevention (3,4). To improve the response, the Training Program in Epidemiology Applied to Services of the Brazilian National Health System was established in 2000, training professionals to investigate and control public health events that required support from federal health service management (4). At the same time, the Rapid Emergency Response Center was created, strengthening the management of outbreaks and epidemics (5).

With the creation of the Health Surveillance Secretariat (6) in 2003, replacing the National Epidemiology Center, national actions were structured. Hospital Epidemiological Surveillance (7,8) was created in 2004 to improve the reporting and investigation of diseases, especially communicable diseases, with the support of the Hospital Epidemiology Centers, which became part of the National Hospital Epidemiological Surveillance Network in 2021 (9). The Vigidesastres program (10,11), established in 2005, is based on management of risks associated with disasters, including risk reduction actions (prevention, preparedness and monitoring), disaster management (alert, communication and response) and recovery (rehabilitation and reconstruction) from disaster exposure, with 52 focal points distributed throughout Brazil (12).

The revision of the International Health Regulations (13) in 2005 led to the creation of National Focal Points in signatory countries for communication with the World Health Organization. In 2006, the Health Surveillance Secretariat became the focal point (14) in Brazil and, in the same year, the National Strategic Health Surveillance Information Center was created, responsible for implementing the International Health Regulations and communicating about public health emergencies (15). In 2007, the National Public Health Emergency Alert and Response Network (CIEVS Network) was structured to expand these capacities nationally (16).

Ordinance No. 2952/2011 (17) regulated, within the SUS, declaration of Public Health Emergencies of National Concern, used in situations of outbreaks, epidemics, disasters or insufficient of health care that require urgent prevention, control and mitigation measures, with a coordinated national response. 

**Figure 1:**
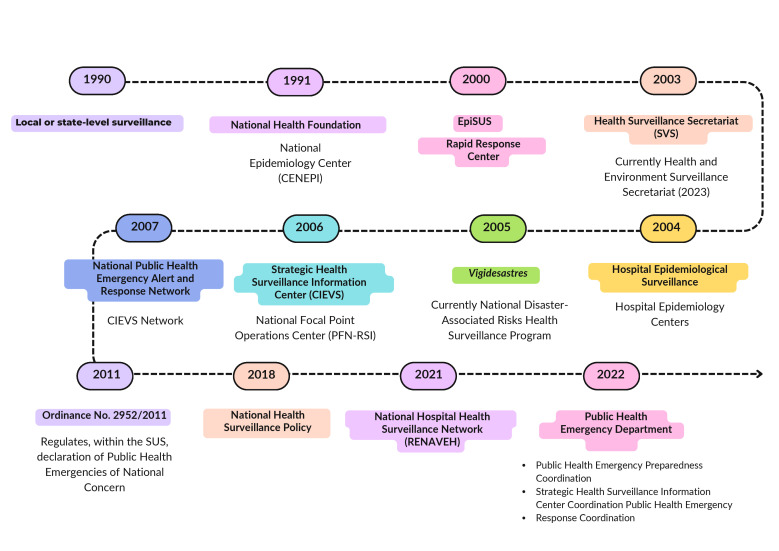
Timeline of the implementation of public health emergency management. Brazil, 2024

The 2018 National Health Surveillance Policy (18) emphasizes the importance of cooperation between different spheres of health service management to strengthen capacity for preparedness, surveillance and response to public health emergencies, ensuring efficient and coordinated actions to prevent, control and mitigate the impacts of these emergencies. In 2022, the creation of the Public Health Emergency Department, currently structured into three coordinations, strengthened the national coordination of these actions, acting through a surveillance network articulated at all levels of management (19) (Figure 1).

## Public Health Emergency Management 

Addressing public health emergencies requires preparedness, surveillance and response actions, which should occur in an interconnected and complementary manner. With regard to preparedness, the Training Program in Epidemiology Applied to SUS Services plays a central role, offering training at fundamental, intermediate and advanced levels (4). The Public Health Emergency Training Program complements this initiative, promoting continuing education and simulations to improve response skills and identify vulnerabilities in contingency plans (20).

Contingency plans have a structuring function, guiding procedures and responsibilities for responding to emergencies. At the federal level, the Public Health Emergency Response Plan has as its principle the use of a coordination and control system (21). In 2024, the Contingency Plan Preparation Guide was launched with the aim of standardizing these plans throughout the SUS (22). There are also contingency plans for emergencies caused by chemical, biological, radiological and nuclear agents, natural disasters, epidemics and outbreaks, among others (23).

Another essential action is the logistics of strategic inputs. This action ensures the maintenance of health services and the most effective response during emergencies (24) (Table 1).

**Table 1:** Lines of action: preparation, surveillance and response. Brazil, 2024

Lines	Actions
**Line I**: Preparedness	EpiSUS is a training program in epidemiology applied to SUS services, with a predominance of practical activities, focusing on epidemiological reasoning, data analysis, detection, response and communication. Characterized as a pyramidal system, offered at three levels (advanced, intermediate and fundamental), in addition to providing training for tutors in field epidemiology.
	The Public Health Emergencies Training Program promotes training and enhancement of professionals working in health services through continuing and permanent education, on the three levels of SUS management - preparation, surveillance and response to public health emergencies.
	Contingency plans aim to guide the organizational structure, operational procedures and responsibilities for responding to emergencies, based on a decision-making roadmap.
	Simulations and other types of training serve to assess the organizational capacity of contingency plans, thus allowing identification of vulnerabilities and gaps within the response system.
	Medicines and strategic supplies are bought and stored in order to maintain essential health services. These kits must be sent within 24 hours, depending on the emergency, and must consist of medicines and strategic supplies with the capacity to serve up to 500 people for a period of three months.
**Line II**: Surveillance	The National Strategic Health Surveillance Information Center (*CIEVS Nacional*) aims to detect, verify, evaluate, monitor and communicate rumors and events that may constitute health emergencies of national concern.
	The CIEVS network, made up of decentralized CIEVS units in the states and municipalities, acts as a sentinel for public health events, expanding the capacity for early detection of emergencies. These centers are integrated by technology and information and communication, thus allowing a coordinated response.
	The Vigidesastres program monitors, for example, the number of homeless, displaced and affected people and deaths; municipalities in states of alert or calamity; and events secondary to the disaster, such as trauma and infectious diseases that require the development of a contingency plan.
	Health surveillance actions in environmental and epidemiological monitoring of events resulting from chemical, biological, radiological and nuclear agents.
	Hospital Epidemiology Centers detect health problems or notifiable diseases through active searches in strategic locations in hospitals, such as the emergency room, inpatient units, laboratories and outpatient clinics. Other important resources in the hospital are the pharmacy, the medical records service and the anatomic pathology laboratory.
	The Event Monitoring Committee discusses and assesses events of public health concern with the participation of technical areas - at the federal level. This committee meets weekly and carries out actions aimed at integrating, reviewing, sharing and discussing all monitored events.
	The Integrated Joint Health Operations Centers are used in mass events to monitor public health emergencies, increasing the capacity for integrated responses between the three spheres of government.
	Voluntary External Evaluation carried out by the WHO is intended to assess the country’s capacity to prevent, detect and respond quickly to risks to public health, whether natural or resulting from deliberate or accidental events.
	The National Epidemiological Intelligence Center is made up of a multidisciplinary team, with technical and scientific support, using advanced methods and robust analyses to access and integrate health data. It aims to integrate epidemiological analysis capacity into evidence-based decision making.
**Line III**: Response	The Emergency Operations Center acts as a coordination structure, facilitating decision-making and ensuring the execution of actions and efficient communication during emergencies. Its characteristics include centralized decision-making and flexibility, as it is possible to adapt its functions to the size of the response required for each type of emergency.
	Risk Communication and Community Engagement involves the exchange of clear, transparent and contextualized information about risks, with the aim of influencing informed behavior and decisions on the part of the population during an emergency.
	Increased financial resources to fund the response to emergencies within the scope of SUS primary and specialized health care and health surveillance.
	Rapid response teams during emergencies are made up of trained specialists and act as a task force that can be quickly mobilized. Vigidesastres, EpiSUS (advanced) and the SUS National Force are examples of teams that, among other functions, also act as rapid response teams during emergencies.

Within the scope of surveillance, the CIEVS Network is a strategic monitoring structure made up of units spread across states, strategic municipalities, capitals and border regions, in addition to indigenous health districts. The network acts as a sentinel for detecting emergencies, evaluating events under the criteria of the International Health Regulations (16,25). These events, classified as unexpected, impacting public health, or with potential for spreading internationally, may be reported to the World Health Organization.

The CIEVS Network uses event-based surveillance strategies, which consider formal and informal sources, and indicator-based surveillance, which analyzes data from national information systems (16). The Vigidesastres program complements surveillance actions, monitoring impacts of disasters (12). Surveillance of chemical, biological, radiological and nuclear factors was strengthened in 2023, with the creation of a specific technical area for monitoring these factors.

The Hospital Epidemiology Centers support the CIEVS Network in reporting and investigation of hospital deaths, in addition to monitoring outbreaks and epidemics through the National Hospital Surveillance Network (7).

The Event Monitoring Committee coordinates the analysis and discussion of events of public health concern. Large mass events are monitored by the Integrated Health Joint Operations Center, strengthening the response capacity between the three spheres of government. Brazil also carries out voluntary external evaluations to monitor basic surveillance and response capabilities.

The National Epidemiological Intelligence Center is being structured, which will integrate epidemiological models and quantitative forecasts into surveillance strategies. This move forward promises to strengthen early warning systems and optimize the capacity to respond to public health emergencies, ensuring more agile and informed action throughout the national territory (26) (Table 1).

Among response actions, Emergency Operations Centers are fundamental for emergency management, using the Operations Command System to organize resources and actions. Emergency Operations Centers are characterized by the ability to lead, coordinate and support the response to an event that has implications for the health of the population or health services (27). 

At the national level, these Centers are triggered when a public health emergency exceeds local or state response capacity (17). This can also be used to prepare and forestall the emergency response, even if it has not yet been confirmed in Brazilian territory. Examples of action include the COVID-19 pandemic, the Yanomami crisis, disasters such as Brumadinho and situations of forced migration. Although migration does not always constitute a public health emergency, health surveillance has recognized its relevance, creating specific groups for monitoring it (28).

The work of the Emergency Operations Centers requires transparency through reports and records, which are essential for engaging authorities, health service managers, health professionals and the population. Risk communication and community engagement are essential elements in this process (29). In 2024, the increase in financial resources was regulated to support responses to emergencies in the SUS, covering primary and specialized care and health surveillance. These resources aim to strengthen actions, reducing impacts on the population’s health and health services (30).

Rapid response teams, such as those of the Vigidesastres Program, the Training Program in Epidemiology Applied to the SUS (advanced) and the SUS National Force, play a crucial role in emergencies. These teams are made up of trained specialists and act as a task force that can be quickly mobilized (Table 1). 

Addressing public health emergencies in Brazil has become more complex, demanding effective and coordinated responses throughout the national territory. Since the creation of the SUS, several strategies have been implemented to improve preparedness, surveillance and response to emergencies, focusing on intersectorality and the involvement of all spheres of health service management.

Guaranteeing specific financial resources for emergencies is essential to enable quick and coordinated actions, providing greater agility in implementing measures during crises and reinforcing the health system’s response capacity.
