# Supplementary material for: History of the implementation of public health emergency management in Brazil
Source: Epidemiol Serv Saude. 2025 May 2;34:e20240498. doi: 10.1590/S2237-96222024v34e20240498.en (PMC12052327; doi:10.1590/S2237-96222024v34e20240498.en)
Supplement: Translation

# Translation: Histórico de implementação da gestão às emergências em saúde pública no Brasil

**DOI:** 10.1590/S2237-96222024v34e20240498.pt

**Authors:** Ana Sara Semeão de Souza, João Roberto Cavalcante, Raquel Proença, Igor de Assis Rodrigues, Carlos Henrique Michiles Frank, Daniel Roberto Coradi de Freitas, Edenilo Baltazar Barreira, Ethel Leonor Maciel, Márcio Henrique de Oliveira Garcia

**Affiliations:** 1 Ministério da Saúde, Secretaria de Vigilância em Saúde e Ambiente, Brasília, DF, Brasil

**Keywords:** Vigilância em Saúde Pública, Capacidade de Resposta ante Emergências, Sistema Único de Saúde, Declaração de Estado de Emergência

## Abstract

As emergências de saúde pública podem causar impactos significativos nas esferas social, econômica e de saúde. Para mitigar esses efeitos, abordagens e programas em níveis nacionais e globais têm sido desenvolvidos, com foco na preparação, vigilância e resposta, especialmente para emergências de importância internacional (1). Como Estado-membro da Organização Mundial da Saúde e signatário de seus acordos e suas convenções, o Brasil possui o compromisso de garantir a execução de ações em saúde com foco nas emergências em saúde pública. Apesar de experiências bem-sucedidas na gestão de emergências, esse ainda é um campo emergente, com limitada produção científica. Este artigo descreve o histórico da gestão das emergências em saúde pública no nível federal e suas estratégias na preparação, vigilância e resposta a esses eventos no Sistema Único de Saúde.

## Introdução

A Fundação Nacional de Saúde (2), criada em 1991, desempenhou papel essencial ao integrar o Centro Nacional de Epidemiologia para promover o uso da epidemiologia na formulação de políticas de saúde no Sistema Único de Saúde (SUS). Nos anos 1990, a resposta às emergências era descentralizada e dependia de apoio externo, como o dos Centros de Controle e Prevenção de Doenças dos Estados Unidos (3,4). Para aprimorar a resposta, o Programa de Treinamento em Epidemiologia Aplicada aos Serviços do Sistema Único de Saúde foi instituído em 2000, capacitando profissionais para investigar e controlar eventos de saúde pública que exigiam apoio da gestão federal (4). Simultaneamente, foi criado o Núcleo de Respostas Rápidas em Emergências, fortalecendo a gestão de surtos e epidemias (5).

Com a criação da Secretaria de Vigilância em Saúde (6) em 2003, substituindo o Centro Nacional de Epidemiologia, foram estruturadas ações nacionais. A Vigilância Epidemiológica em Âmbito Hospitalar (7,8) foi criada em 2004 para melhorar a notificação e investigação de agravos, especialmente doenças transmissíveis, com suporte dos Núcleos Hospitalares de Epidemiologia, que passaram a integrar a Rede Nacional de Vigilância Epidemiológica Hospitalar em 2021 (9). O Vigidesastres (10,11), instituído em 2005, se fundamenta na gestão de riscos associados a desastres, incluindo ações de redução do risco (prevenção, preparação e monitoramento), manejo do desastre (alerta, comunicação e resposta) e recuperação (reabilitação e reconstrução) à exposição de desastres, com 52 pontos focais distribuídos pelo Brasil (12).

A revisão do Regulamento Sanitário Internacional (13) em 2005 levou à criação de Pontos Focais Nacionais nos países signatários para comunicação com a Organização Mundial de Saúde. Em 2006, a Secretaria de Vigilância em Saúde tornou-se o ponto focal (14) no Brasil e, no mesmo ano, foi criado o Centro de Informações Estratégicas de Vigilância em Saúde, responsável pela implementação do Regulamento Sanitário Internacional e comunicação sobre emergências de saúde pública (15). Em 2007, a Rede Nacional de Alerta e Resposta às Emergências em Saúde Pública (Rede CIEVS) foi estruturada para expandir essas capacidades nacionalmente (16).

A Portaria n º 2.952/2011 (17) regulamentou, no SUS, a declaração de Emergência em Saúde Pública de Importância Nacional, utilizada em situações de surtos, epidemias, desastres ou desassistência que demandem medidas urgentes de prevenção, controle e mitigação, com resposta nacional coordenada. 

A Política Nacional de Vigilância em Saúde de 2018 (18) enfatiza a importância da cooperação entre diferentes esferas de gestão para fortalecer a capacidade de preparação, vigilância e resposta a emergências em saúde pública, garantindo ações eficientes e articuladas para prevenir, controlar e mitigar os impactos dessas emergências. Em 2022, a criação do Departamento de Emergência em Saúde Pública, atualmente estruturado em três coordenações, fortaleceu a coordenação nacional dessas ações, atuando por meio de uma rede de vigilância articulada em todos os níveis de gestão (19) (Figura 1).

**Figura 1:** Linha do tempo do histórico de implementação da gestão de emergências em saúde pública. Brasil, 2024

## Gestão de Emergências em Saúde Pública

O enfrentamento de emergências em saúde pública requer ações de preparação, vigilância e resposta, que ocorrem de maneira interligada e complementar. No que diz respeito à preparação, o Programa de Treinamento em Epidemiologia Aplicada ao SUS desempenha papel central, oferecendo formação em níveis fundamental, intermediário e avançado (4). O Programa de Formação em Emergências em Saúde Pública complementa essa iniciativa, promovendo educação continuada e simulados para aprimorar habilidades de resposta e identificar vulnerabilidades em planos de contingência (20).

Os planos de contingência têm função estruturante, orientando procedimentos e responsabilidades para resposta às emergências. Em nível federal, o Plano de Resposta a Emergências de Saúde Pública tem como princípio a utilização de sistema de coordenação e controle (21). Em 2024, foi lançado o Guia para Elaboração de Planos de Contingência visando padronizar esses planos em todo o SUS (22). Existem também planos de contingência para emergências por agentes químico, biológico, radiológico e nuclear, desastres naturais, epidemias e surtos, entre outros (23).

Outra ação essencial é a logística de insumos estratégicos. Essa ação assegura a manutenção dos serviços de saúde e a resposta mais eficaz durante emergências (24) (Table 1).

**Tabela 1:** Eixos de ação: preparação, vigilância e resposta. Brasil, 2024

Eixos	Ações
**Eixo I**: Preparação	EpiSUS é um programa de treinamento em epidemiologia aplicada aos serviços do SUS, com predominância de atividades práticas, enfoque no raciocínio epidemiológico, análise de dados, detecção, resposta e comunicação. Caracterizado como um sistema piramidal, ofertado em três níveis (avançado, intermediário e fundamental), além de contar com a formação de tutores em epidemiologia de campo.
	O Programa de Formação em Emergências em Saúde Pública promove capacitação e o aperfeiçoamento de profissionais que estão no serviço por meio da educação continuada e permanente, que atuam nos três níveis de gestão do SUS – preparação, vigilância e resposta às emergências em saúde pública.
	Planos de contingência visam nortear a estrutura organizacional, os procedimentos operacionais e as responsabilidades para a resposta às emergências, tendo como base um roteiro para a tomada de decisão.
	Simulados e outros tipos de treinamentos servem para avaliar a capacidade operacional dos planos de contingência, permitindo assim a identificação de vulnerabilidades e lacunas dentro do sistema de resposta.
	Aquisição e armazenamento de medicamentos e insumos estratégicos destinam-se a manutenção de serviços de saúde essenciais. Esses kits devem ser enviados em até 24 horas, dado a emergência e, deve ser composto por medicamentos e insumos estratégicos com capacidade para atender até 500 pessoas por um período de três meses.
**Eixo II**: Vigilância	O Centro Nacional de Informações Estratégicas em Vigilância em Saúde (CIEVS Nacional) tem como finalidade detectar, verificar, avaliar, monitorar e comunicar r rumores e eventos que possam constituir emergências em saúde pública de importância nacional.
	A Rede CIEVS composta por unidades CIEVS descentralizadas nos estados e municípios, atua como sentinela para eventos em saúde pública, ampliando a capacidade de detecção precoce de emergências. Estes centros são integrados por tecnologia de informação e comunicação, permitindo uma resposta coordenada.
	O Vigidesatres realiza monitoramento, por exemplo, do número de desabrigados, desalojados, afetados e óbitos; os municípios em estado de alerta ou calamidade; e eventos secundários ao desastre, como trauma e doenças infecciosas que exigem a elaboração de plano de contingência.
	Ações de vigilância em saúde no monitoramento ambiental e epidemiológico de eventos decorrentes de agentes químico, biológico, radiológico e nuclear.
	Núcleos Hospitalares de Epidemiologia detectam agravos ou doenças de notificação compulsória a partir da busca ativa em locais estratégicos no hospital, como o pronto-socorro, unidades de internação, laboratório e ambulatório. Outras fontes importantes no hospital são a farmácia, o serviço de arquivo médico e o laboratório de anatomia-patológica.
	O Comitê de Monitoramento de Eventos discute e avalia os eventos de interesse à saúde pública com a participação das áreas técnicas – a nível federal. Esse comitê se reúne semanalmente e realiza ações visando à integração, revisão, compartilhamento e discussão de todos os eventos monitorados.
	Centro Integrado de Operações Conjuntas da Saúde, são utilizados em grandes eventos de massa para o monitoramento de emergências em saúde pública aumentando a capacidade de respostas integradas entre as três esferas de governo.
	Avaliação Externa Voluntária realizada pela OMS destina-se a avaliar as capacidades do país em prevenir, detectar e responder rapidamente aos riscos para a saúde pública, sejam eles naturais ou resultantes de eventos deliberados ou acidentais.
	Centro Nacional de Inteligência Epidemiológica é composta por equipe multidisciplinar, com apoio técnico e científico, utiliza métodos avançados e análises robustas para acessar e integrar dados de saúde. Visa a ampliação da capacidade de análise epidemiológica na tomada de decisão baseada em evidências.
**Eixo III**: Resposta	O Centro de Operação de Emergência atua como uma estrutura de coordenação, facilitando a tomada de decisões e garantindo a execução de ações e a comunicação eficiente durante emergências. Têm como características a centralização da tomada de decisões e a flexibilidade, pois é possível adaptar suas funções ao tamanho da resposta necessária para cada tipo de emergência.
	Comunicação de risco e Engajamento Comunitário envolve a troca de informações claras, transparentes e contextualizadas sobre riscos, com o objetivo de influenciar comportamentos e decisões informadas por parte da população durante uma emergência.
	Incremento de recursos financeiros para custeio de resposta às emergências no âmbito da atenção à saúde primária e especializada, e da vigilância em saúde do SUS.
	Equipes de resposta rápida durante as emergências são formadas por especialistas treinados e atuam como uma força-tarefa que pode ser rapidamente mobilizada. O Vigidesatres, o EpiSUS (avançado) e a Força Nacional do SUS são exemplos de equipes que, entre outras funções, também atuam como equipes de resposta rápida durante as emergências.

No âmbito da vigilância, a Rede CIEVS é uma estrutura estratégica de monitoramento composta por unidades espalhadas por estados, municípios estratégicos, capitais e regiões de fronteira, além de distritos sanitários indígenas. A rede atua como sentinela para detectar emergências, avaliando eventos sob os critérios do Regulamento Sanitário Internacional (16,25). Esses eventos, classificados como inesperados, de impacto à saúde pública, ou com potencial de propagação internacional, podem ser notificados à Organização Mundial de Saúde.

A Rede CIEVS utiliza estratégias de vigilância baseada em eventos, que considera fontes formais e informais, e vigilância baseada em indicadores, que analisa dados de sistemas nacionais de informação (16). O Vigidesastres complementa as ações de vigilância, monitorando impactos de desastres (12). A vigilância de fatores químicos, biológicos, radiológicos e nucleares foi fortalecida em 2023, com a criação de área técnica específica para monitoramento desses fatores.

Os Núcleos Hospitalares de Epidemiologia apoiam a Rede CIEVS na notificação e investigação de óbitos hospitalares, além de monitorarem surtos e epidemias por meio da Rede Nacional de Vigilância Hospitalar (7).

O Comitê de Monitoramento de Eventos coordena a análise e discussão de eventos de interesse à saúde pública. Grandes eventos de massa são acompanhados pelo Centro Integrado de Operações Conjuntas da Saúde, fortalecendo a capacidade de resposta entre as três esferas de governo. O Brasil realiza também avaliações externas voluntárias para monitorar capacidades básicas de vigilância e resposta.

Está sendo estruturado o Centro Nacional de Inteligência Epidemiológica, que integrará modelos epidemiológicos e previsões quantitativas às estratégias de vigilância. Esse avanço promete fortalecer sistemas de alerta precoce e otimizar a capacidade de resposta a emergências em saúde pública, garantindo a atuação mais ágil e informada em todo o território nacional (26) (Tabela 1).

Entre as ações de resposta, os Centros de Operações de Emergências são fundamentais na gestão das emergências, utilizando o Sistema de Comando de Operações para organizar recursos e ações. O Centro de Operações de Emergências é caracterizado pela capacidade de liderar, coordenar e apoiar a resposta a um evento com implicações para a saúde da população ou para os serviços de saúde (27). 

No nível nacional, esse centro é ativado quando uma emergência em saúde pública ultrapassa a capacidade de resposta local ou estadual (17). Este também poderá ser utilizado para preparar e antecipar a resposta à emergência, mesmo que ainda não tenha sido confirmada em território brasileiro. Exemplos de atuação incluem a pandemia de covid-19, a crise Yanomami, desastres como o de Brumadinho e situações de migração forçada. Apesar de as migrações nem sempre configurarem como emergência em saúde pública, a vigilância em saúde tem reconhecido sua relevância, criando grupos específicos para seu monitoramento (28).

A atuação do Centro de Operações de Emergências exige transparência por meio de informes e registros, fundamentais para engajar autoridades, gestores, profissionais de saúde e a população. A comunicação de risco e o engajamento comunitário são elementos essenciais nesse processo (29). Em 2024, foi regulamentado o aumento de recursos financeiros para apoiar respostas às emergências no SUS, abrangendo atenção primária, especializada e a vigilância em saúde. Esses recursos visam fortalecer ações, reduzindo impactos na saúde da população e nos serviços de saúde (30).

Equipes de resposta rápida, como o Vigidesastres, o Programa de Treinamento em Epidemiologia Aplicada ao SUS (avançado) e a Força Nacional do SUS, desempenham papel crucial nas emergências. Essas equipes são formadas por especialistas treinados e atuam como força-tarefa que pode ser rapidamente mobilizada (Tabela 1). 

O enfrentamento das emergências em saúde pública no Brasil tem se tornado mais complexo, demandando respostas eficazes e coordenadas em todo o território nacional. Desde a criação do SUS, diversas estratégias têm sido implementadas para aprimorar a preparação, vigilância e resposta às emergências, com foco na intersetorialidade e no envolvimento de todas as esferas de gestão.

A garantia de recursos financeiros específicos para emergências é essencial para viabilizar ações rápidas e coordenadas, proporcionando maior agilidade na execução de medidas durante crises e reforçando a capacidade de resposta do sistema de saúde.
